# Optimization of Quinoa‐Based Gluten‐Free Bread Production Using Microbial Transglutaminase Enzyme and Hydroxypropyl Methyl Cellulose (HPMC) by Response Surface Methodology

**DOI:** 10.1002/fsn3.70891

**Published:** 2025-09-01

**Authors:** Zahra Ghodosipoor, Omid Zahed, Hossein Fallahzadeh, Neda Mollakhalili‐Meybodi, Amene Nematollahi

**Affiliations:** ^1^ Student Research Committee, Department of Food Science and Technology, School of Public Health Shahid Sadoughi University of Medical Sciences Yazd Iran; ^2^ Research Center of Food Hygiene and Safety, Department of Food Science and Technology, School of Public Health Shahid Sadoughi University of Medical Sciences Yazd Iran; ^3^ Center for Healthcare Data Modeling, Departments of Biostatistics and Epidemiology, School of Public Health Shahid Sadoughi University of Medical Sciences Yazd Iran; ^4^ Department of Food Safety and Hygiene, School of Health Fasa University of Medical Sciences Fasa Iran

**Keywords:** hydroxypropyl methylcellulose, microbial transglutaminase enzyme, quinoa, technological characteristics

## Abstract

Celiac is an autoimmune disorder that is triggered by gluten proteins. Bread is the staple foodstuff in which gluten networks play a detrimental role. This study investigated the optimization of transglutaminase (TG) and hydroxypropyl methylcellulose (HPMC) using Response Surface Methodology (RSM) and central composite design (CCD) to simulate the functionality of gluten proteins in quinoa‐based gluten‐free bread. The concentrations of TG and HPMC varied within the ranges of 0%–1.5% w/w and 0%–2% w/w, respectively. A model was ultimately developed using the CCD and the specified range values for the two factors, with measurements taken for specific volume (SV), moisture content (MC), overall acceptance (OA), and hardness. The study identified optimal values of 0.414 g/100 g for TG and 1.283 g/100 g for HPMC, achieving specific values of 2.320 cm^3^/g for SV, 39.161% for MC, 7.454 for OA, and 5431.121 g for hardness, with a desirability of 0.983. To verify the presented model, the bread quinoa sample produced with formula (F1) was evaluated and then compared with the control samples (quinoa bread in the absence of TG and HPMC (F2) and wheat bread (F3)) in terms of rheological properties, microstructure, and physicochemical properties. Among the gluten‐free breads (F1 and F2), improvement in all parameters has been achieved through the incorporation of TG and HPMC. This improvement demonstrates the effectiveness of TG and HPMC to well mimic the functional characteristics of gluten in gluten‐free breads. Despite more uniform distribution of air bubbles at F1, its higher MC and lower SV compared to F3 verified its lower water migration through the baking process.

## Graphical abstract

The optimized bread formulation demonstrated improved rheological properties, microstructure, and physicochemical qualities, with a more uniform gas distribution than conventional wheat bread. However, it showed reduced water migration during baking, a notable difference from wheat bread, which could affect shelf stability and requires further study.

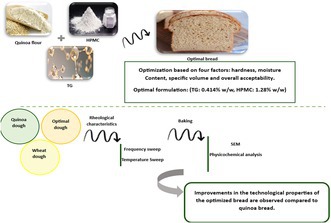

## 1 Introduction

Celiac disease is an autoimmune disorder that affects approximately 1% of the world's population (King et al. 2020) and is triggered by gluten proteins present in wheat, rye, and barley (Caio et al. 2019). Patients with celiac disease exhibit a wide range of symptoms, including abdominal pain, diarrhea, constipation, and/or nutrient malabsorption. To date, the only effective treatment for celiac disease is a strict gluten‐free diet (Aljada et al. 2021). The main gluten‐free sources that can potentially be used in food formulations include cereals (rice, corn, and sorghum), small grains (fonio, sorghum, and millet), and pseudocereals (buckwheat, quinoa, and amaranth) (Kaur et al. 2024).

According to the CODEX STAN 118‐1979 standard, a product is classified as gluten‐free if it either contains no wheat, rye, barley, or oats, or has a gluten content of less than 20 mg/kg (Bustamante et al. 2017). Bread, cakes, pasta, biscuits, fermented cereal‐based drinks, and ready‐made soups are the main foodstuffs in which concerns existed about the presence of gluten. Bread is the staple foodstuff produced and consumed all around the world, with a vital role of gluten in its structure forming (Woomer and Adedeji 2021). The gluten network, stabilized by intra‐ and intermolecular disulfide bonds, plays a key role in dough rheology by providing elasticity, resistance to stretching, mixing tolerance, and the ability to retain gases (Anton and Artfield 2008). In the absence of gluten, bakery products often exhibit undesirable characteristics, such as poor texture, unappealing color, low volume, and inferior aroma and flavor (Esposito et al. 2024). Additionally, gluten‐free products are prone to rapid staling, crumb hardening, crust softening, and moisture migration (Carini et al. 2017).

Quinoa (*Chenopodium quinoa Willd*), a well‐known gluten‐free pseudocereal, is rich in high‐quality protein. It is considered a rich source of lysine, methionine, and histidine, which are the main limiting amino acids in many cereals, including wheat (Jamali et al. 2024). It is also a rich source of fiber, minerals (e.g., calcium, iron, zinc and magnesium), vitamin E, and antioxidants (Zare et al. 2022). Considering quinoa's valuable nutritional characteristics, growing interest has existed for using it in formulations suitable for the consumption of people with gluten intolerance (Azizi et al. 2020). However, gluten‐mimicking agents need to be added.

Starches (Horstmann et al. 2017), low‐lactose dairy derivatives (Úbeda et al. 2023), hydrocolloids (Mir et al. 2016), enzymes (Scherf et al. 2018) and prebiotics (Marasco et al. 2020) are the main constituents used to improve the structure, mouthfeel, acceptability, and shelf life of gluten‐free bakery products, and their potential has been investigated in different studies. These treatments are expected to have a synergistic effect, especially when a single treatment alone is not sufficient to improve the technological properties of the final product. This study is done to investigate the combined use of hydrocolloids and enzymes.

The microbial transglutaminase (MTG) enzyme, with the ability to create a covalent crosslink between the carboxyl group of glutamine and the amino group of lysine, is potentially able to strengthen the product by affecting the protein's solubility (Mostafa 2020). In other words, the crosslink formation induced by MTG not only preserves the essential amino acid lysine but also causes the formation of protein polymers with water retention capacity, thereby improving the rheological and technological properties of the product (Sulaiman et al. 2022). However, its level needs to be optimized as the excessive use of MTG may lead to the increased density of the bread, which prevents the growth of gas cells and consequently lowers specific volume (Redd et al. 2024).

On the other hand, using gums which are potentially able to trap water and create a gluten‐like structure with desirable viscoelastic properties has been investigated in several studies (Zhao et al. 2021). One of the most widely used gums in the food and bakery industries is hydroxypropyl methylcellulose (HPMC) (Srikanlaya et al. 2022). However, its quantity needs to be optimized to prevent the moist nature of the final product. Regarding Response Surface Methodology (RSM) as an efficient statistical approach, it can be used to minimize the difficulties of the conventional experimentation (Malekjani and Jafari 2020).

Considering the importance of gluten‐free bread formulation for celiac patients, the valuable nutritional properties of quinoa, and the necessity of simulating the technological properties of gluten in bakery products, optimizing the quantities of MTG and HPMC gum in quinoa‐based gluten‐free bread formulation using RSM is recommended.

## 2 Material and Methods

### 2.1 Bread Ingredients

The whole quinoa flour (Quinothin, Yazd, Iran), salt (Semnan, Iran), active dry yeast (Narmila, Iran), oil (Behshahr, Iran), microbial transglutaminase (Aryagostar, Iran) and hydroxyl propyl methyl cellulose (with molecular weight: 240208 Da, donated by Shimi Daroui Darupakhsh Co., Tehran, Iran) were obtained. All chemicals were purchased from Merck and Sigma.

### 2.2 Bread Preparation

Quinoa‐based gluten‐free bread formulations were optimized using central composite design (CCD) and Response Surface Methodology (RSM). The study focused on two key variables: microbial transglutaminase (MTG) concentration (0%–1.5% w/w) and hydroxypropyl methylcellulose (HPMC) content (0%–2% w/w), with ranges established through preliminary testing and literature review.

**FIGURE 1:**
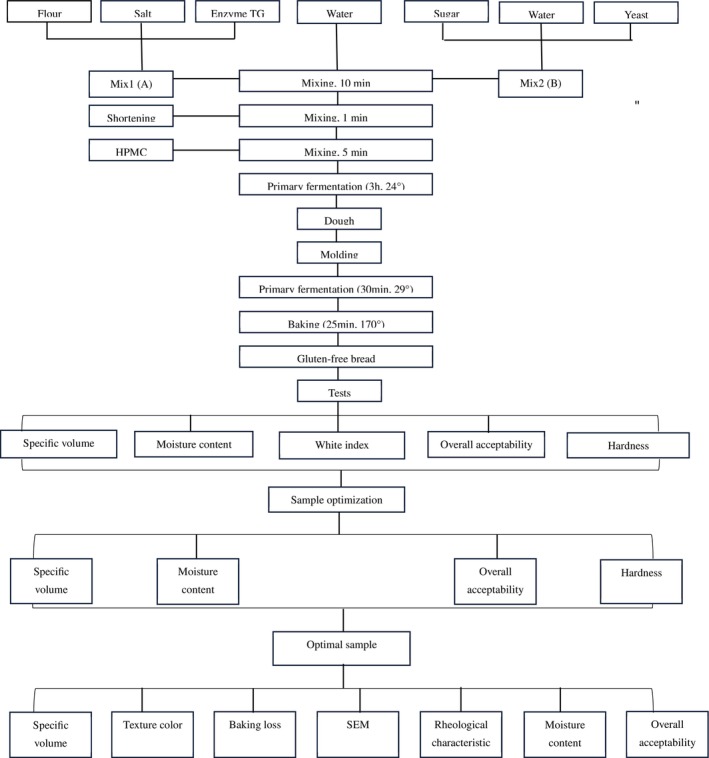
Bread preparation method used in the present study.

The other raw materials are used at fixed content on the basis of 100 g of quinoa flour. Regarding sugar, salt, shortening, and active dry yeast are used at 10%, 1%, 10%, and 2.2% w/w respectively. The quantity of water in each formulation was set using a rheometer (Anton Paar MCR301, GmbH) adjustment to reach an optimum dough consistency. The formulation of bread samples is prepared as mentioned below (as demonstrated at Figure 1). Firstly, the quinoa flour was sieved and mixed with other ingredients: MTG and salt thoroughly (A). About 40% of the required water, active dry yeast, and sugar were mixed and kept for 10 min at laboratory environment (B). The mixtures of A and B were then mixed with the remaining water and kneaded for 10 min. After initial mixing, shortening was added and mixed for 1 min. Then, HPMC was added manually to the mixture and the dough was kneaded at high speed for 5 min. The prepared dough was placed in the fermentation chamber for 3 h at 29°C. The dough was then kneaded for 1 min and evenly distributed into pans. The pans were then incubated at 29°C and 85% humidity for 30 min. The bread was baked for 25 min at 170°C. Once baked, the bread samples were cooled at room temperature and stored in polyethylene bags at 25°C until testing (Shiri et al. 2021).

### 2.3 Evaluation of Bread Baking and Dough Performance

#### 2.3.1 Specific Volume

The specific volume (SV) of quinoa‐based gluten‐free breads was determined on the basis of the rapeseed displacement method. SV was calculated by dividing the loaf volume (cm^3^) of each bread piece by its weight (g) (AACC Method 10–05.01). Measurements were taken 1 h after the bread was removed from the oven (Shiri et al. 2021).

#### 2.3.2 Moisture Content

Regarding, a piece of the bread was placed in an air oven set at 105°C ± 0.05°C, and kept until the weight difference of two measurement intervals of 15 min was less than 0.1% *W*/*W*. The moisture contents were determined as follows:

$$\mathrm{MC}=\frac{W1-W2}{W1}\times 100$$

Where, *W*1 and *W*2 represent the initial weighting and after drying weigh of samples respectively (Rybicka et al. 2019).

#### 2.3.3 Overall Acceptability

30 semi‐trained panelists (female: male ratio of 50: 50 aged between 18 and 45 years old were chosen from the school of public health, Shahid Sadoughi university of medical sciences, Yazd, Iran). Sensory evaluations were conducted according to the 9‐point hedonic scale assessment. Panelists were asked to rate the overall acceptability of the samples on a scale from 1 to 9 in which 1, 5, and 9 represented the lowest, moderate, and highest acceptability as perceived by the panelists, respectively. Good health, willingness to participate, and regular consumption of pan breads were considered as inclusion criteria for panelists to participate (Shivapour et al. 2020).

#### 2.3.4 Color Analysis

Color analyses of bread samples were conducted using the Ultra Scan UV/Vis spectroscopy (Hunter lab, Germany). Regarding the crumb color of each formulation, it was determined and results were reported in terms of lightness (L*), redness (a*) and blueness (b*) (Shukla et al. 2023).

#### 2.3.5 Hardness

The textural characteristics of quinoa‐based gluten free breads were analyzed using a texture profile analyzer (TA20, KOOPA) following the AACC Approved Method 74‐09 (2000). Regarding bread crumbs (20 × 20 × 25 mm) were analyzed by a two‐bite compression test using the 43 mm cylinder probe at a test speed of 1 mm/s with a 50% compression level, a relaxation time of 10 s, a loading cell of 5 kg, and a temperature of 25°C ± 3°C. The hardness of bread samples was determined by measuring the maximum force required during the first bite (Ahlborn et al. 2022).

#### 2.3.6 Baking Loss

The baking loss through the baking process is monitored as follows:

$$\text{Baking loss}\;\left(\%\right)=\left[\left(W_{d}–W_{b}\right)/W_{d}\right]\times 100$$

Where, $W_{d}$ and $W_{b}$ are weights of dough and bread (after baking and cooling respectively) (Turkut et al. 2016).

#### 2.3.7 Microstructure

The microstructures of dried quinoa‐based gluten free samples were determined by scanning electron microscope (SEM) at an accelerating voltage of 15 kV. The samples were coated with a thin layer of gold prior to imaging to enhance their conductivity and improve the image quality (Demirkesen et al. 2013).

#### 2.3.8 Fundamental Rheological Characteristics

The rheological characteristics of quinoa‐based gluten‐free dough samples were evaluated in terms of frequency sweep and temperature sweep tests using a controlled shear/stress rheometer (Anton Paar MCR301, GmbH) with parallel plate geometry. Strain sweep tests were first conducted to identify the viscoelastic region (LVE). Following this, frequency sweep tests were done at a frequency range of1 to 1000 Hz at a constant strain of 0.01%. During these tests, the elastic modulus (*G*′), viscous modulus (*G*″), damping factor (tan *δ*) and complex modulus (G*) were measured and determined as follows;

$$\tan\;\text{34𝛿}=\frac{G^{"}}{G^{\prime }}$$

$$G*=\sqrt{\left(G\prime^{2}+G{"}^{2}\right)}$$

Also, temperature sweep test was done at the certain strain of 0.1% and dough samples were heated in the range of 30°C–90°C. The increasing ratio of temperature was set at 4°C/min (Iacovino et al. 2024).

### 2.4 Response Surface Methodology Optimization

Response Surface Methodology (RSM) is a collection of statistical techniques used for designing experiments, constructing models, evaluating the effects of various factors, and determining the optimal conditions to achieve the best responses. RSM is particularly effective for optimizing processes involving multiple factors in product development. A combination of factors that leads to an optimal response can be identified using design factors and response surface methodology (RSM) (Marcin et al. 2016). In this study, Central Composite Design (CCD) using RSM was employed to accurately predict the optimum conditions of quinoa‐based gluten‐free bread production and to minimize the number of experimental trials. All factors were tested at five levels (Table 1). The experimental ranges for the two significant variables—MTG content (0%–1.5% w/w) and HPMC content (0%–2% w/w) for the CCD trials are presented in Table 1.

**TABLE 1:** Main process variables and 13 trials of a central composite design to investigate the impact of main and interaction effects on optimizing quinoa bread production.

RUN	Independent variables^b^	
	MTG^a^ (% w/w)	HPMC^a^ (% w/w)
1	0.75	1
2	0.75	1
3	1.5	1
4	0.75	1
5	0.75	1
6	0	1
7	0.75	0
8	1.28033	0.292893
9	0.21967	1.70711
10	0.75	1
11	0.21967	0.292893
12	1.28033	1.70711
13	0.75	2

^a^ MTG and HPMC are abbreviations for microbial transglutaminase enzyme and hydroxyl propyl methyl cellulose, respectively.

^b^ Response variables assessed at this study are specific volume (Y_1_), moisture (Y_2_), overall acceptability (Y_3_), and hardness (Y_4_) (as demonstrated at Table 2).

### 2.5 Statistical Analysis

The results were analyzed using Design Expert software (Version 12, Stat‐Ease Inc., USA) and response surface plots were created. Each experiment was repeated three times for accuracy. The data were analyzed using analysis of variance (ANOVA) and are presented as the mean value ± standard deviation (SD) from independent experiments on various samples. Data processing was done using SPSS statistical software (SPSS Statistics 23.0, Chicago, IL, USA). In general, *p*‐values ≤ 0.05 were considered statistically significant.

## 3 Results and Discussion

We used a central composite design (CCD) within the Response Surface Methodology (RSM) framework to develop predictive models and optimize the bread formulation. Thirteen experimental runs were carried out, focusing on four key response parameters: specific volume (SV), moisture content (MC), overall acceptability (OA), and hardness. Table 2 outlines the experimental ranges for the two primary variables (MTG and HPMC concentrations). The ANOVA results (Table 3) revealed that all model terms with *p*‐values < 0.05 were statistically significant. Quadratic models were found to be the best fit for all response variables (*p* < 0.05), with non‐significant Lack of Fit tests (*p* ≥ 0.05), indicating that the models were adequate. Considering the effects of TG on response variables, it was revealed that TG level significantly impacted SV and MC (*p* < 0.05) but had no statistically significant effect on OA and hardness (*p* ≥ 0.05). Similarly, HPMC levels significantly influenced SV, MC, and hardness (*p* < 0.05) but did not significantly affect OA (*p* ≥ 0.05). No significant interaction effect was found between TG and HPMC for any of the investigated parameters (*p ≥ 0.05*).

**TABLE 2:** Technological properties of different quinoa‐based gluten‐free breads as suggested by mixture experimental design.

RUNs	Response variables			
	SV (cm^3^/g)	MC (%)	OA	Hardness (g)
1	2.03	38.8655	7.66	6940.49
2	2.026	39.194	7.66	6790.32
3	1.89	41.5945	6.5	6739.6
4	2.205	38.8768	7.33	7040.07
5	2.087	38.897	7.66	7240.7
6	2.5977	38.3703	6.33	5470.14
7	1.8473	38.2482	6.16	13451.8
8	1.7463	40.9205	7	11034.9
9	2.2836	40.4359	7.16	4892.19
10	2.1045	39.7049	8	5742.1
11	1.9444	38.1229	6.5	8905.88
12	2.3163	41.922	6.66	4170.9
13	2.466	41.2108	6.5	3474.8

**TABLE 3:** Statistical data for the analysis of variance (ANOVA) of the full quadratic model in the optimization of quinoa bread production.

Response variables	Source	Sum of squares	df	Mean square	*F*	*p*
SV	Model	0.6827	7	0.0975	9.08	0.0137
	A‐TG	0.2504	1	0.2504	23.32	0.0048
	B‐HPMC	0.1914	1	0.1914	17.82	0.0083
	AB	0.0133	1	0.0133	1.24	0.3161
	A^2^	0.0139	1	0.0139	1.30	0.3061
	B^2^	9.604E−06	1	9.604E−06	0.0009	0.9773
	A^2^B	0.0001	1	0.0001	0.0136	0.9116
	AB^2^	0.0872	1	0.0872	8.12	0.0358
	Residual	0.0537	5	0.0107		
	Lack of fit	0.0326	1	0.0326	6.16	0.0680
	Pure error	0.0211	4	0.0053		
MC	Model	20.36	5	4.07	25.57	0.0002
	A‐TG	9.78	1	9.78	61.40	0.0001
	B‐HPMC	7.04	1	7.04	44.21	0.0003
	AB	0.4300	1	0.4300	2.70	0.1443
	A^2^	2.19	1	2.19	13.75	0.0076
	B^2^	1.31	1	1.31	8.25	0.0239
	Residual	1.11	7	0.1592		
	Lack of fit	0.5941	3	0.1980	1.52	0.3383
	Pure error	0.5205	4	0.1301		
OA	Model	3.81	5	0.7613	8.15	0.0078
	A‐TG	0.0072	1	0.0772	0.0774	0.7889
	B‐HPMC	0.0802	1	0.0802	0.8586	0.3850
	AB	0.2500	1	0.2500	2.68	0.1458
	A^2^	1.80	1	1.8	19.31	0.0032
	B^2^	2.12	1	2.12	22.67	0.0021
	Residual	0.6536	7	0.0934		
	Lack of fit	0.4291	3	0.1430	2.55	0.1940
	Pure error	0.2245	4	0.0561		
Hardness	Model	8.774E+07	5	1.755E+07	45.41	0.0001
	A‐TG	1.282E+06	1	1.282E+06	3.32	0.1113
	B‐HPMC	7.805E+07	1	7.805E+07	201.94	< 0.0001
	AB	2.031E+06	1	2.031E+06	5.26	0.0556
	A^2^	7.631E+05	1	7.631E+05	1.97	0.2028
	B^2^	5.002E+06	1	5.002E+06	12.94	0.0088
	Residual	2.705E+06	7	3.865E+05		
	Lack of fit	1.327E+06	3	4.422E+05	1.28	0.3941
	Pure error	1.379E+06	4	3.447E+05		

Technological characteristics of quinoa‐based gluten free breads (in terms of SV, MC, OA, and hardness) as functions of independent variables (TG and HPMC level coded as A and B respectively) are presented in Table 4 based on four equations (*Y*_1_–*Y*_4_).

**TABLE 4:** Second‐order quadratic models developed for the response variables.

Response variables^a^	Second order quadratic model	Regression coefficients	
		$R^{2}$	$R_{\mathrm{adj}}^{2}$
SV	Y_1_ = 2.09−A 0.2502 + B 0.2187 + AB 0.0577 + A^2^ 0.0448 + B^2^ 0.0012 + A^2^B 0.0086 + AB^2^0.2089	0.9271	0.8250
MC	Y_2_ = 39.11 + A 1.11 + B 0.938−AB 0.3279 + A^2^ 0.561 + B^2^ 0.4345	0.9481	0.9110
OA	Y_3=_ 7.66 + A 0.0301 + B 0.1001−AB 0.25−A^2^ 0.5091−B^2^ 0.5516	0.8535	0.7488
H	Y_4=_ 6750.74 + A 400.38−B 3123.41−AB 712.58−A^2^ 331.21 + B^2^ 848.00	0.9701	0.9487

^a^ Response variables assessed in this study include specific volume (Y1), moisture content (Y2), overall acceptability (Y3), and hardness (Y4), as well as the levels of TG% w/w and HPMC% w/w, which are represented in coded units A and B, respectively.

Numerical optimization was done to determine the precise values of independent variables that would yield the desired responses. Regarding the SV, MC, OA, and hardness were assessed together, and the first solution was selected in this study. The software suggested optimal values of 0.414 and 1.283% w/w for TG and HPMC. These values provided the following responses: SV of 2.320 cm^3^/g, MC of 39.161%, OA of 7.454, and hardness of 5431.121 g, with a desirability score of 0.983.

### 3.1 Validation of the Optimized Formulation

The bread was prepared using the optimized levels of TG and HPMC suggested by the model. The actual results for specific volume (SV), moisture content (MC), overall acceptability (OA), and hardness were compared with the values predicted by the software (Table 5). A one‐sample *t*‐test revealed no significant differences between the actual and predicted values (*p* ≥ 0.05), confirming the model's accuracy and validating the optimization process. The optimized formulation (designated as F1) was then evaluated against two control samples (F2: Quinoa‐based control (without TG or HPMC) and F3: Conventional wheat‐based dough). This comparison looked at key technological properties to evaluate how the optimized recipe performed against both gluten‐free and standard wheat‐based benchmarks.

**TABLE 5:** Results of the verification test for the optimal point with estimated values in the model.

Responses	Predicted results	Experimental results	*p*
Specific volume (cm^3^/g)	2.320	2.339 ± 0.05	0.22
Moisture content (%)	39.161	38.937 ± 0.01	0.69
Overall acceptability	7.454	7.512 ± 0.17	0.29
Hardness (g)	5431.121	5462.11 ± 32.62	0.73

### 3.2 Technological Characteristics of Optimized Formulation

The optimized formulation (F1) was developed and its technological characteristics were compared with control samples (quinoa dough and bread without TG and HPMC, i.e., (F2)) and wheat dough and bread, i.e., (F3) in terms of rheological characteristics, microstructure, and physicochemical characteristics.

#### 3.2.1 Rheological Characteristics

##### 3.2.1.1 Frequency Sweep

**FIGURE 2:**
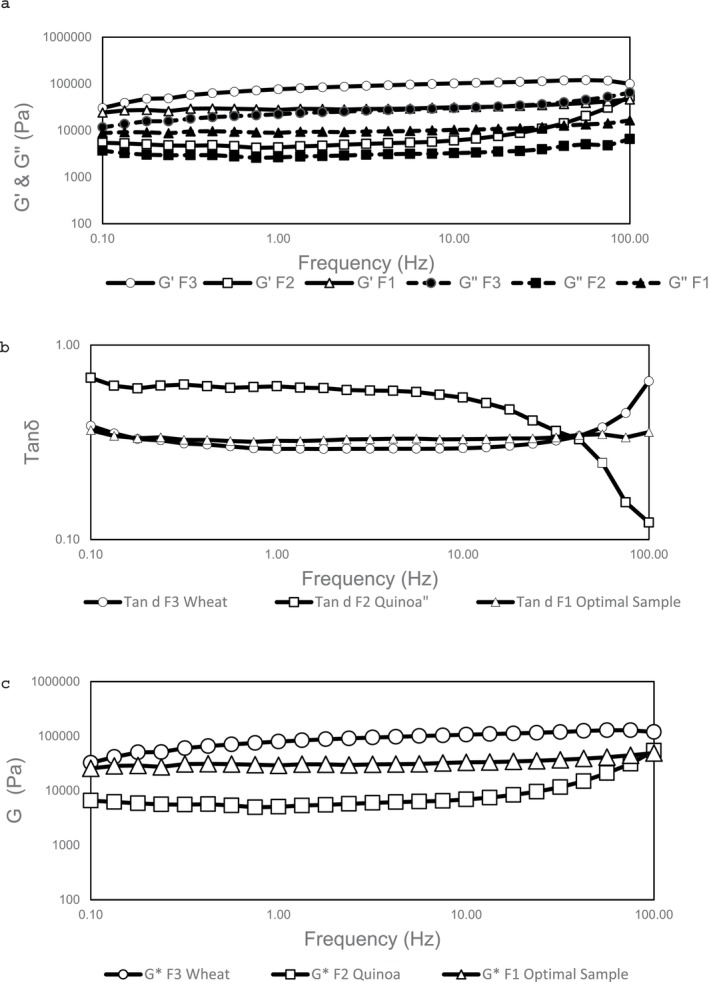
Frequency sweep ((a) Storage and loss modulus (*G*′ and *G*″), (b) Damping factor (tan *δ*), and (c) Complex modulus (G*)) test of optimized formulation in comparison to control samples. F1: Optimized formulation, F2: Quinoa dough in the absence of TG and HPMC, F3: Wheat dough.

The frequency sweep test, a fundamental oscillatory rheological measurement, was used to evaluate the time‐dependent viscoelastic behavior of formulations. This test provides critical insights into the microstructure of the final product (Zhang et al. 2024). Conducted within the linear viscoelastic (LVE) region, where the system exhibits reversible viscoelastic behavior, the test measures four key parameters: storage modulus (*G*′), loss modulus (*G*″), complex modulus (G*), and damping factor (tan *δ*). As shown in Figure 2a, both *G*′ and *G*″ exhibit frequency‐dependent behavior, with *G*′ remaining dominant across the entire frequency spectrum. This indicates the formation of a stable, elastic gel‐like network (Yazar and Demirkesen 2023). The highest elastic modulus is observed in F3 (wheat dough), while the lowest is found in F2 (quinoa‐based dough without TG and HPMC). Considering the complex modulus (G*) and damping factor (tan *δ*) (Figure 2b,c), the optimized formulation (F1) is placed between the two control samples (F2 and F3). However, the highest complex modulus is found in F3, attributed to the unique 3D structure of the gluten network. A significant increase in G* of F1 compared to F2 indicates that the incorporation of TG and HPMC in the quinoa‐based gluten‐free dough formulation can mitigate the poor structure that typically fails to retain gases (Yazar and Demirkesen 2023). The elevated complex modulus (G*), reflecting improved dough stability, appears to result from the synergistic effect of TG and HPMC, which simultaneously enhance both the elastic (*G*′) and viscous (*G*″) moduli. This demonstrates their combined impact on the material's viscoelastic properties. A decrease in the damping factor of F1 compared to F2 declares further enhancement of its elastic modulus, which aligns with the findings of (Mollakhalili‐meybodi et al. 2023). It seems that the crosslink formation induced by the microbial transglutaminase enzyme alters the viscoelastic properties of the dough, as previously noted by (Han et al. 2013). The isopeptide bonds formation between proteins catalyzed by the transglutaminase enzyme provides a robust protein network within the dough, improving its rheological characteristics (da Silva Ramos et al. 2023). Additionally, HPMC gum has the ability to interact with both the aqueous and non‐aqueous phases of the dough, leading to a more uniform and stable structure (Hager and Arendt 2013).

##### 3.2.1.2 Temperature Sweep

**FIGURE 3:**
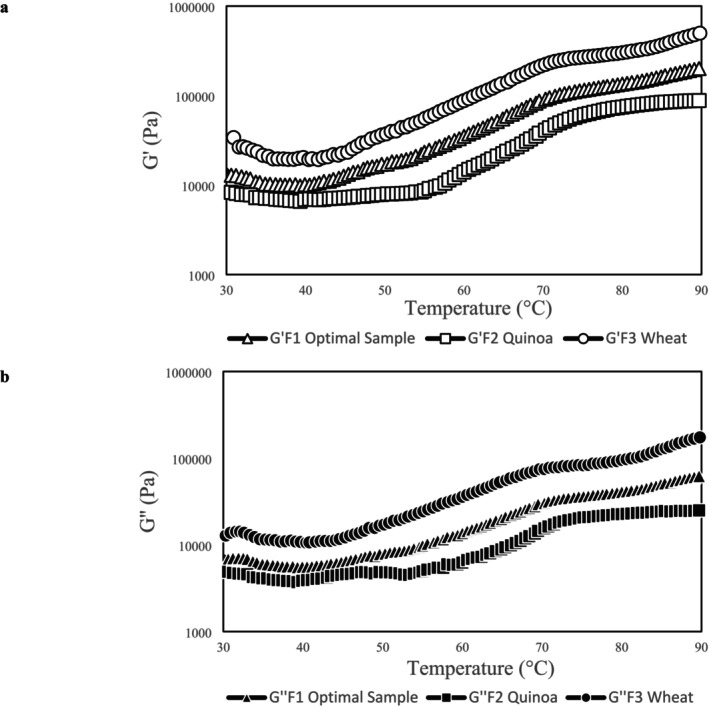
Temperature sweep ((a) storage modulus (*G*′) and loss modulus (*G*″)) test of optimized formulation in comparison to control samples. F1: Optimized formulation, F2: Quinoa dough in the absence of TG and HPMC, F3: Wheat dough.

Temperature sweep test is done to precisely examine how the structural and viscoelastic characteristics of dough change through the baking process (Hager and Arendt 2013). As the dough bakes, gel formation creates a three‐dimensional network, leading to an increase in both the storage modulus (*G*′) and loss modulus (*G*″). These rheological measurements were performed at a fixed strain of 0.1% while heating the dough samples from 30°C to 90°C. As illustrated in Figure 3, all samples exhibited predominantly elastic behavior, with *G*′ consistently higher than *G*″ across the entire temperature range. The elastic modulus exhibited a two‐phase response: first, a gradual decline followed by a subsequent increase as the temperature rose. The initial decrease in *G*′ reflects dough softening, which occurs due to enhanced polymer mobility and structural reorganization at elevated temperatures (Cappelli et al. 2020).

Although the samples showed similar trends in thermal behavior, significant differences were observed in their gelatinization temperatures–defined as the temperature at which the elastic modulus (*G*′) begins to rise. This variation in gelatinization behavior between quinoa and wheat flours aligns with previous findings by Chakraborty et al. (2022).

As demonstrated in Figure 3, a significant decrease in gelatinization temperature is observed with the addition of TG and HPMC, dropping to 40°C compared to 55°C for F1 and F2 respectively. Hydrocolloids inclusion in formulation is supposed to potentially lower the gelatinization temperature on the basis of their types and interaction with other components (Shan 2023). Differences in both storage modulus (*G*′) and Loss modulus (*G*″) were also evident across the various formulations. Specifically, the highest *G*′ and *G*″ were recorded for F3 (i.e., wheat dough), while the lowest were found at F2 (quinoa‐based gluten free bread without TG and HPMC). The changes observed during thermal processing are influenced by factors such as water content, protein and polysaccharide level and their ratio and interactions (Iacovino et al. 2024). Initial heating softens the dough, which is attributed to protein weakening. The subsequent sharp increase in the elastic modulus is driven by protein network crosslinking and starch gelatinization (Ahmed 2015). The improvement in the rheological characteristics of quinoa‐based gluten free bread with the inclusion of TG and HPMC is believed to result from both physical and chemical mechanisms. Physically, HPMC enhances water absorption as reported by (Nammakuna et al. 2016), while chemically, TG promotes crosslinking within the protein network (Diowksz and Sadowska 2021).

#### 3.2.2 Scanning Electron Microscopy (SEM) Analysis

**FIGURE 4:**
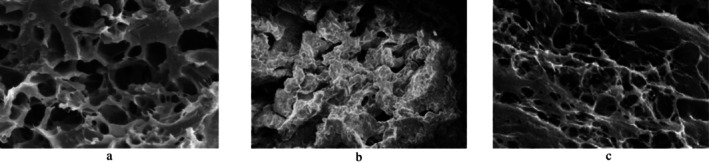
Scanning electron microscopy analysis of: (a) Optimal bread, (b) Quinoa bread, (c) wheat bread.

Digital image analysis is used to evaluate the qualitative characteristics of baked goods, e.g., by monitoring the integrity and distribution of air bubbles throughout the entire matrix (Srikanlaya et al. 2022; Venkatesan and Shivaani 2024). In gluten‐free formulations, the absence of a coherent and uniform gluten network results in an inability to restore gases through baking. Regarding qualitative characteristics, optimized gluten‐free bread samples compared to control ones (i.e., quinoa bread and wheat bread) were determined using image analysis at a magnification of 500×. As evident in Figure 4b, the lack of three‐dimensional structures in gluten‐free bread without additive (i.e., F2) leads to the settling of flour particles and prevents the entrapment of air bubbles. In contrast, the inclusion of TG and HPMC in the formulation (i.e., F1) resulted in a higher number of air bubbles with a more uniform distribution (Figure 4a). The incorporation of the microbial transglutaminase enzyme has been proved to form new covalent crosslinks, mimicking the function of a 3D gluten network (Meybodi et al. 2019). However, its incorporation must be optimized, as an excessively strong network is unappealing in bread formulation as it limits protein flexibility and restricts oven expansion (Min et al. 2025; Moradi et al. 2021). The more uniform distribution of gases in F1 compared to wheat bread is attributed to differences in its formulation (e.g., particle size, crystallinity, amylose content, fiber content, water absorption content and ratio) (Huang et al. 2022). The presence of fiber has been previously stated to decrease starch‐protein binding, resulting in a more homogeneous crumb (Demirkesen et al. 2013). The protective effects of HPMC in creating a more porous structure in gluten‐free bread and consequently increasing its specific volume are in accordance with (Villanueva et al. 2024).

#### 3.2.3 Physicochemical Analysis

The physicochemical characteristics of optimized formulation (F1) were compared to the control samples (F2 and F3) in terms of specific volume (SV), crumb color analysis (L*, a* and b*), baking loss, moisture content, and overall acceptability, as shown in Table 6.

**TABLE 6:** A comparative analysis of the physicochemical properties of the optimized formulation and the control samples.

Samples	Parameters						
	SV (cm^3^/g)	*L* *	*a* *	*b* *	Baking loss (%)	Moisture content (%)	Overall acceptability
F1	2.34 ± 0.05^b^	73.22 ± 0.08^c^	10.01 ± 0.01^a^	17.09 ± 0.07^a^	6.30 ± 0.02^b^	38.94 ± 0.01^b^	7.55 ± 0.12^b^
F2	1.78 ± 0.07^c^	75.13 ± 0.01^b^	10.13 ± 0.07^a^	16.62 ± 0.02^a^	10.62 ± 0.01^c^	44.18 ± 0.08^a^	6.23 ± 0.14^c^
F3	2.91 ± 0.13^a^	87.19 ± 0.03^a^	8.21 ± 0.03^b^	11.45 ± 0.09^b^	8.67 ± 0.06^a^	36.12 ± 0.11^c^	8.99 ± 0.01^a^

*Note:* Evaluated sample produced with formula (F1) and control samples quinoa bread in the absence of TG and HPMC (F2) and wheat bread (F3).

* Different lower letters in each column indicate a statistically significant difference (*p* < 0.05).

The specific volume (SV), which indicates the volume expansion of bread, ranged between 1.78 and 2.91cm^3^/g. The lowest and highest content is observed at F2 (i.e., gluten free quinoa bread in the absence of TG and HPMC) and F3 (i.e., wheat bread) respectively. The inclusion of TG enzyme and HPMC in the optimized formulation (F1) increased its SV by approximately 31.4% compared to F2. This suggests that the quality defects induced by the lack of a 3D network in gluten free pseudocereals were mitigated. The specific volume of breads greatly depends on the ability of its protein network to restore gases produced during fermentation and kneading (Shiri et al. 2021). Although there was no significant difference *(p ≥ 0.05)* in the volume of the optimized bread compared to wheat bread, the significantly lower SV (*p* < 0.05) of F1 can be attributed to its higher weight. Both TG and HPMC had a significant impact (*p* < 0.05) on the SV of gluten‐free bread, as shown in Table 6. The crosslinking induced by the TG enzyme likely facilitated network formation, enabling better gas retention (Moore et al. 2006; Morreale et al. 2018). Additionally, HPMC is known to form a network through its hydrophobic functional groups able to wrap the batter constituents (Culetu et al. 2021; Meybodi et al. 2019; Morreale et al. 2018).

Regarding the moisture content (MC), it has been revealed that the optimized formulation containing TG and HPMC has an intermediate MC of 38.94%. F2 exhibited a significantly higher (*p* < 0.05) MC compared to F1. The water‐solid interactions facilitated by hydrocolloids, e.g., HPMC, have been stated to hinder water migration through baking (Carini et al. 2017; Guan et al. 2023; Mariotti et al. 2013). This assumption is supported by the significantly lower baking loss observed in F1 compared to F3.

In terms of crumb color analysis, the optimized formulation (F1) and control samples (F2 and F3) showed decreased lightness and increased a* and b* values compared to F3. The decreased lightness in quinoa‐containing samples is attributed to the original darker color of quinoa flour (Codina et al. 2017). Gas entrapment in bread formulations enhances light scattering, which can reduce lightness (Bartkiene et al. 2023). Thus, the significantly decreased lightness observed in F1 compared to F2 is likely due to its higher specific volume and enhanced light scattering.

When comparing overall acceptability, wheat bread (F3) was found to be more appealing, which was expected since wheat bread is more commonly consumed by the panelists. However, among the gluten‐free breads, the optimized formulation (F1) showed a reasonable improvement in overall acceptability. This improvement is thought to result from enhanced textural characteristics and mouthfeel perception, as TG and HPMC have been confirmed as texture modifiers in this study (Encina‐Zelada et al. 2019).

## 4 Conclusions

This study successfully refined a quinoa‐based gluten‐free bread recipe using response surface methodology (RSM), incorporating microbial transglutaminase (TG; 0.414% w/w) and hydroxypropyl methylcellulose (HPMC; 1.283% w/w). The results demonstrate that TG and HPMC effectively mimic gluten's functional qualities, significantly improving the bread's technological characteristics. Specifically, the optimized formulation exhibited enhanced rheological properties, an improved microstructure, and superior physicochemical attributes. Notably, the optimized bread showed a more even gas distribution compared to traditional wheat bread. However, while these improvements are promising, the reduced water migration during baking—the key difference from wheat bread—could affect shelf stability and warrants further investigation.

## Author Contributions

**Zahra Ghodosipoor:** data curation (equal), formal analysis (equal), methodology (equal), writing – original draft (equal). **Omid Zahed:** project administration (equal), supervision (equal), writing – review and editing (equal). **Hossein Fallahzadeh:** investigation (equal), methodology (equal), writing – original draft (equal). **Neda Mollakhalili‐Meybodi:** project administration (equal), supervision (equal), writing – review and editing (equal). **Amene Nematollahi:** project administration (equal), supervision (equal), writing – review and editing (equal).

## Conflicts of Interest

The authors declare no conflicts of interest.

## Data Availability

The corresponding author can provide the data supporting this study's findings upon reasonable request.
